# Pre-eclampsia: the Potential of GSNO Reductase Inhibitors

**DOI:** 10.1007/s11906-017-0717-2

**Published:** 2017-03-07

**Authors:** Thomas R. Everett, Ian B. Wilkinson, Christoph C. Lees

**Affiliations:** 1 Department of Fetal Medicine, Leeds Teaching Hospitals Trust, Leeds, UK; 2 University of Leeds, Leeds, UK; 3 Experimental Medicine and Immunotherapeutics, Addenbrooke’s Hospital, Box 98, Cambridge, UK; 4 Imperial College London, London, UK; 5 Imperial College Healthcare NHS Trust, London, UK; 6 Department of Development and Regeneration, KU Leuven, Leuven, Belgium

**Keywords:** Pre-eclampsia, S-nitrosoglutathione, GSNO, GSNO reductase inhibitors

## Abstract

**Purpose of Review:**

Pre-eclampsia remains a leading worldwide cause of maternal death and of perinatal morbidity. There remains no definitive treatment except delivery of the fetus.

**Recent Findings:**

Recent insights into the cardiovascular changes that are evident prior to, during, and persist after pre-eclampsia have improved understanding of the underlying pathophysiology—disruption of normal endothelial function and decreased nitric oxide bioavailability. S-nitrosoglutathione (GSNO) is an endogenous S-nitrosothiol that acts as a NO pool and, by replenishing or preventing the breakdown of GSNO, endothelial dysfunction can be ameliorated. GSNO reductase inhibitors are a novel class of drug that can increase NO bioavailability.

**Summary:**

GSNO reductase inhibitors have demonstrated improvement of endothelial dysfunction in animal models, and in vivo human studies have shown them to be well tolerated. GSNOR inhibitors offer a potentially promising option for the management of pre-eclampsia.

## Introduction

Pre-eclampsia is a multisystem disorder, which manifests clinically as hypertension and proteinuria after 20 weeks of pregnancy. Pre-eclampsia occurring at or close to term is usually treatable by delivery with minimal risk to mother or baby. However, in approximately 1% of pregnancies the condition is early onset, and this usually coincides with worse severity, particularly before 32 weeks gestation. In this situation, expeditious conservative management focusing on control of hypertension and seizure prevention to gain fetal maturity is key. Although antihypertensive medication is used, there is no current treatment that targets the underlying pathophysiology [1, 2].

The underlying pathological processes of pre-eclampsia are hypothesized to occur in two stages [3]. Abnormal placentation is suggested to be the initiating event resulting in reduced placental perfusion, in turn, leading to increased oxidative stress, which, in combination with a maternal predisposition, results in endothelial dysfunction. This manifest by changes in a number of signaling pathways and homeostatic mechanisms, but impaired nitric oxide (NO) bioavailability [4] is thought to play a major role in the maternal manifestations of pre-eclampsia such as hypertension and likewise platelet activation, proteinuria, and oedema. More recently, abnormal pre-pregnancy blood pressure has been shown to relate to risk of pre-eclampsia [5], and abnormal arterial function in the first trimester is associated with higher likelihood of PE [6]. So the prevailing wisdom relating to the placenta’s central role in pre-eclampsia is likely to be an over simplification.

## Relationship of Pre-eclampsia and Endothelial Dysfunction

Pre-eclampsia causes disruption of normal endothelial barrier, structure, and function, resulting in a state of endothelial dysfunction which is characterized by decreased NO bioavailability. Arterial and cardiac function are abnormal before and during the disease’s clinical manifestation [7, 8••, 9]. Consequently, there is an increase in vascular tone [10, 11], hypertension [12, 13], increased permeability of the vasculature and resultant proteinuria, and oedema [14, 15]. There is also a shift towards a proinflammatory and prothrombotic state [4, 16], particularly as a result of platelet activation [17].

Women who have had pre-eclampsia are at a higher long-term risk of adverse cardiovascular outcomes including stroke, myocardial dysfunction, and death due to a vascular event [18–21]. Indeed, the major risk factors for pre-eclampsia are those classically associated with endothelial dysfunction and long-term cardiovascular morbidity including systolic hypertension, obesity, diabetes mellitus, and hypercholesterolaemia [22–25]. It is now suggested that there is pre-existing pre-pregnancy endothelial dysfunction in women who go on to develop pre-eclampsia. Studies starting prior to pregnancy and following women throughout pregnancy have allowed an insight into haemodynamic changes in normal pregnancy [26, 27]; abnormal cardiovascular adaptation in early pregnancy may be associated with birth weight [28]. The degree to which these risk factors affect endothelial function pre-pregnancy and the degree to which they are exacerbated by pregnancy and pre-eclampsia are currently under investigation.

Asymmetric dimethylarginine (ADMA) is an endogenous eNOS inhibitor. Levels of ADMA are higher in women at high risk of pre-eclampsia as determined by abnormal uterine artery Doppler waveform [7]. And, in those women who go on to develop pre-eclampsia, there is an inverse correlation of ADMA with FMD, suggesting that increased ADMA may reduce NO bioavailability and thus contribute to the development of pre-eclampsia. FMD is reduced in the early second trimester in women who go on to develop both pre-term and term pre-eclampsia when compared to those who do not become hypertensive or develop gestational hypertension [29].

Decreased bioavailability of NO provides a potential therapeutic target for novel drug therapy of pre-eclampsia. NO donors, used in a research context, reduce blood pressure (BP), and platelet activation in pre-eclampsia whilst having no detrimental effect on placental perfusion [1, 30]. Prophylaxis with glyceryl trinitrate (GTN) in high-risk women reduces overall adverse outcome related to ‘placental syndromes’ though not the incidence of pre-eclampsia itself [2, 31]. However, the utility of most NO donors including GTN is limited by hypotension, side effects, and tachyphylaxis.

**Fig. 1:**
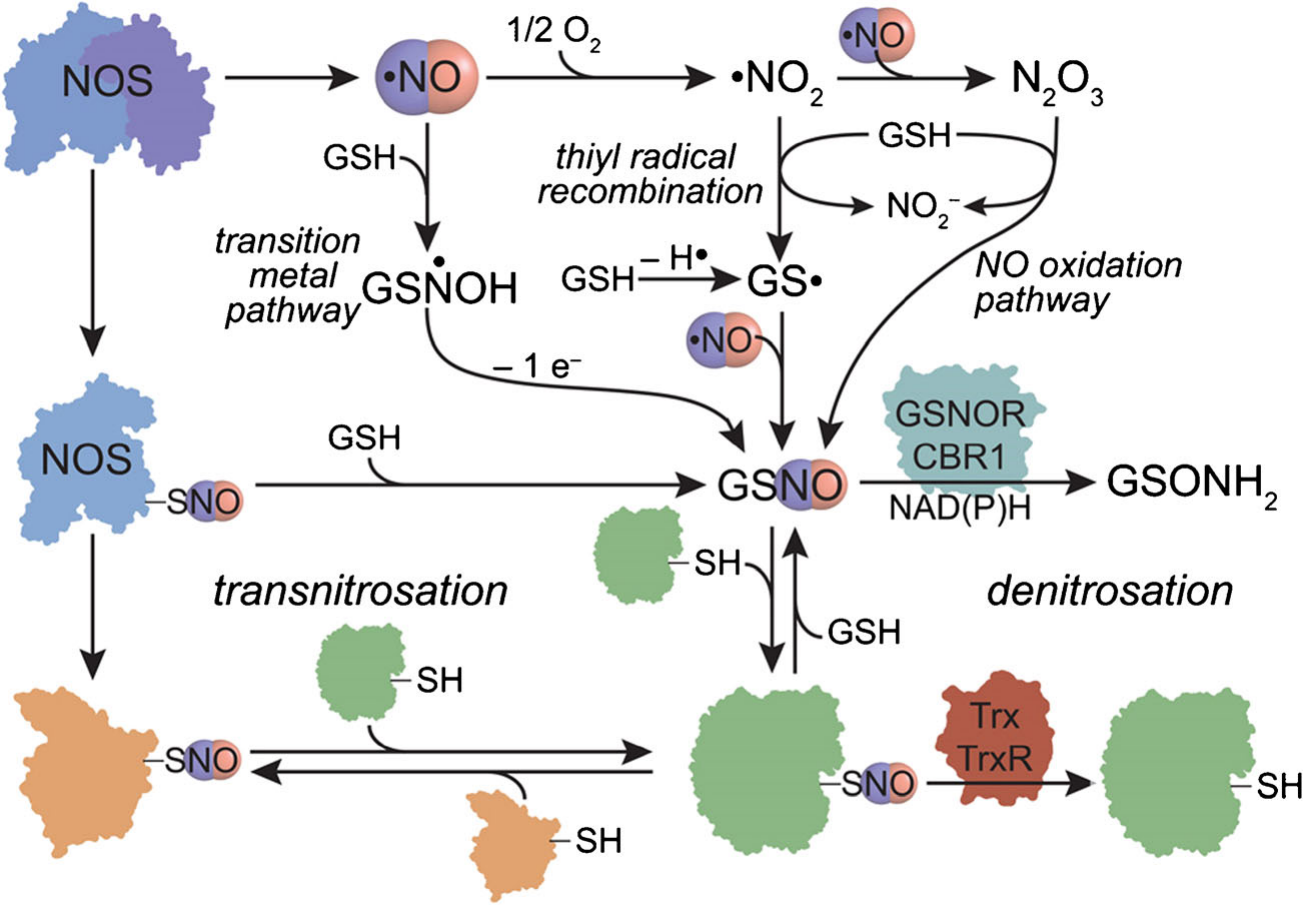
Potential transnitrosation signaling pathways. *NOS* nitric oxide synthase, *NO* nitric oxide, *GSNO* S-nitrosoglutathione, *GSH* glutathione, *GSNOR* S-nitrosoglutathione reductase, *CBR1* carbonyl reductase, *Trx* thioreduxin, *TrxR* thioreduxin reductase. Adapted from Smith et al. [33•]. Reproduced with permission

The primary source of NO is endothelial nitric oxide synthase (eNOS). NO is extremely short-lived and, in order to have more than a transient effect following synthesis, it must be stored in a stable, bioavailable form. The primary receptor of NO is soluble guanylate cyclase (sGC), with subsequent activation of this enzyme and the production of cyclic GMP. cGMP acts as a secondary messenger and is involved in the regulation of multiple intracellular pathways, notably smooth muscle relaxation and platelet inhibition. S-nitrosothiols are a class compounds that have an NO group attached to the thiol (RSH) moiety by a single chemical bond. S-nitrosothiols release the NO moiety by mechanisms including exposure to light, heat, and transition metals, in addition to enzymatic bioactivation. As such, these compounds, particularly GSNO act as a stable intracellular bioavailable NO pool [32]. The precise pathways for cellular nitrosothiol formation and degradation remain unclear, although the following schematic (Fig. 1), adapted from Smith et al. [33•], outlines potential mechanisms. Another potentially valuable property of GSNO is that glutathione acts as a free-radical ‘sink’.

**Fig. 2:**
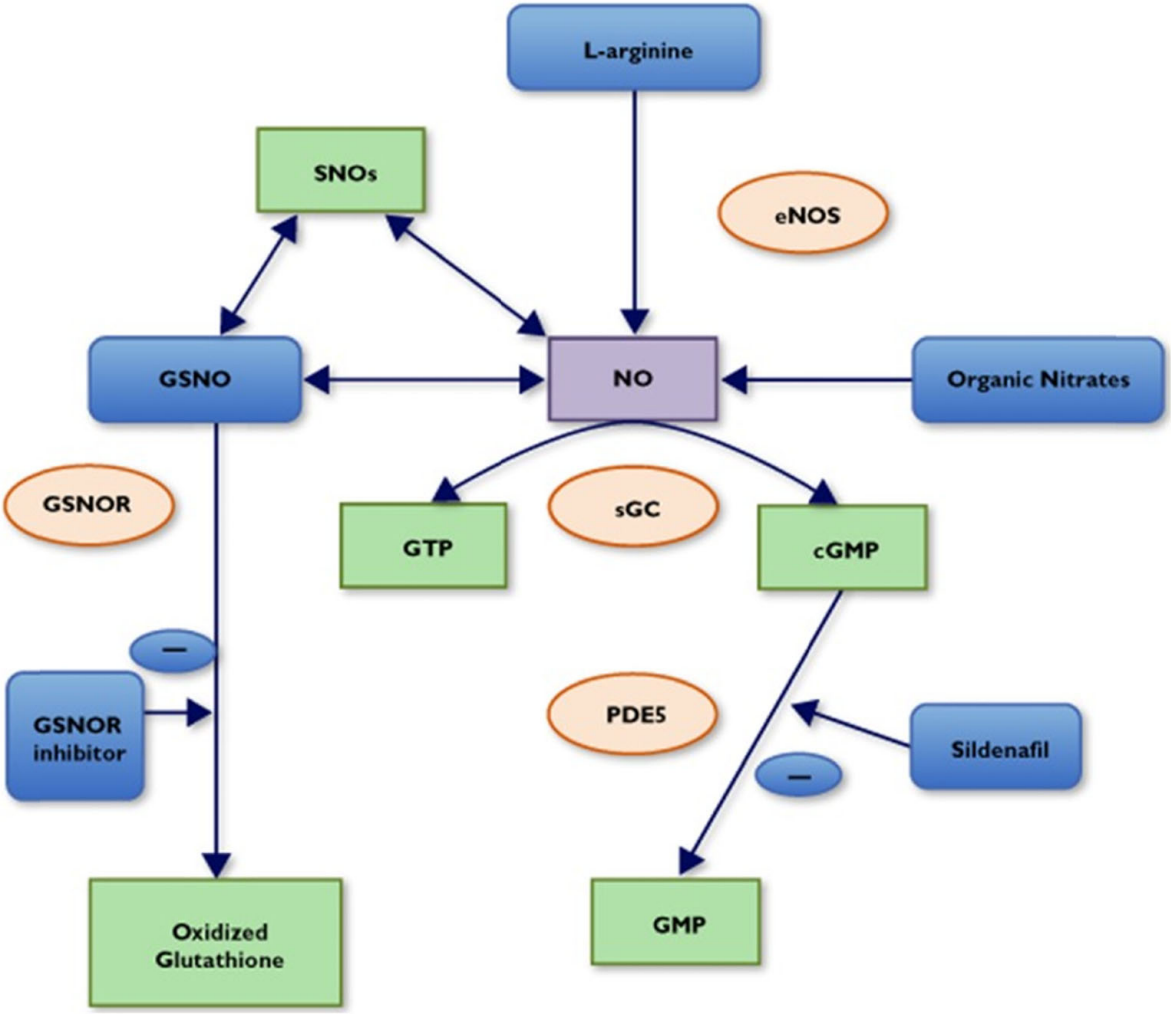
Simplified schematic of NO/S-nitrosothiol pathways and potential therapeutic targets. Reproduced from Johal et al. [34]

Increased levels of GSNO may facilitate NO-mediated processes, whereas increased activity GSNOR (the regulatory enzyme in GSNO catabolism) may impair these processes through reduction of NO bioavailability. It has been proposed that, due to the fact that GSNOR interferes with processes relevant to cardiovascular health, inhibition of GSNOR may be beneficial [32]. The potential sites of therapeutic intervention in the NO pathway are outlined in Fig. 2.

## GSNO in Pre-eclampsia

GSNO was first used over 20 years ago in relation to the treatment of pre-eclampsia in a woman with severe HELLP (haemolysis, elevated liver enzymes and low platelets) syndrome (thought to be an extreme variant of pre-eclampsia) which was refractory to conventional management. Improvements in blood pressure and platelet count were noted almost immediately following commencement of GSNO infusion [35]. Further investigation of the use of GSNO in severe pre-eclampsia showed improvements in blood pressure, uterine artery pulsatility index (which is classically abnormally high in pre-eclampsia), and a reduction in platelet activation [30]. It is also notable that other studies investigating the replenishment of NO, using GTN patches, suggest that improved outcomes and prolongation of pregnancy may be achievable [31, 36, 37].

We have previously performed an in vivo study of GSNO infusion in pre-eclampsia in six women with early-onset pre-eclampsia [38]. GSNO resulted in a reduction in augmentation index, an indirect measure of NO-sensitive small vessel tone, from baseline (*P* < 0.001) with significant changes occurring at infusion rates of 30 and 100 μg/min GSNO (mean reduction in AIx-75: −6 and −13% respectively, *P* < 0.05). Reductions in both diastolic (*P* = 0.017) and systolic central BPs (*P* = 0.008) were also seen. The reduction in central BP was significant at 100 μg/min GSNO (*P* < 0.05). Similarly MAP dose dependently reduced with increasing GSNO infusion rate (*P* = 0.004). Peripheral systolic BP was unchanged, but peripheral diastolic BP fell (*P* = 0.012). No significant changes were found in central or peripheral pulse pressures, and maternal heart rate remained constant.

Platelet surface P-selectin expression, a marker of platelet activation, was lower following GSNO infusion (*P* = 0.03). GSNO infusion in pre-eclampsia may also reduce proteinuria. The urine protein:creatinine ratios were lower in relation to their starting levels at the conclusion of the GSNO infusion.

The fetal heart rate did not change during GSNO infusion, and there were no significant cardiotocograph changes. There were no changes in maternal uterine, fetal umbilical, middle cerebral, or ductus venosus Doppler PI at any dose of GSNO.

Whilst the primary purpose of the study was to test the hypothesis that replacement of NO with different doses of GSNO in early-onset pre-eclampsia would restore arterial function, neonatal, and maternal safety outcomes were collected. The perinatal outcomes were not different from fetuses of similar weight and gestation that were not exposed to GSNO. There were no maternal adverse outcomes, although three women reported headache, which resolved with paracetamol administration.

Further preliminary investigation on the quantification of GSNO in women with severe pre-eclampsia, has suggested that GSNO is found in lower circulating quantities than in women without pre-eclampsia (personal unpublished data).

Although S-nitrosoglutathione has potential as a therapeutic option for pre-eclampsia, there are significant difficulties in its administration. GSNO degrades rapidly when exposed to light and heat so it requires long-term storage in a freezer. After reconstitution, GSNO requires immediate intravenous infusion and needs to be protected from light and kept cool. These factors limit its utility as a treatment option, particularly in the developing world, where the burden of disease and where the availability of reliable refrigeration are often limited.

## GSNO Reductase Inhibitors Identification and In Vivo Studies

Following the increased awareness of GSNO as a major player in NO metabolism and as a repository for NO, which otherwise has a short biological half-life, the role of GSNO reductase was further investigated. Various studies found that there was deregulation of GSNOR leading to lower SNO concentrations and adverse effects on the respiratory, cardiovascular, and gastrointestinal systems. Conversely, mice with a genetic deletion of GSNOR are protected from asthma-like airway hyperresponsivity by the increased SNO concentrations acting as an endogenous bronchodilator. This, in turn, led to a targeted high throughput screening of commercially available compounds by N30 Pharmaceuticals (now Nivalis Therapeutics) (Boulder, CO, USA) to identify compounds that demonstrated potential as GSNOR inhibitors [39•]. Following identification and synthesis selected, compounds were further tested for cytotoxicity. Minimal toxicity was observed towards epithelial lung cells, and a majority of those studied also demonstrated minimal cytochrome P450 inhibition. Similarl no evidence of mutagenicity was found in a bacterial mutagen screen, and in vivo toxicology studies in mice demonstrated that the substances were well tolerated although at the highest doses alterations to liver function tests were found on prolonged exposure [40••].

Intravenous administration of a novel GSNOR inhibitor (N6022), daily for over 7 days showed that it was well tolerated and there were few side-effects. Those that were demonstrated, e.g., cough, nasal congestion, and headache were well tolerated and were not dose-limiting. A further phase 1 study of administration of an orally administered GSNOR inhibitor (N91115) found that it was well tolerated in healthy subjects at up to 2.5 times to expected therapeutic exposure, for over 14 days. Pharmacokinetics studied support; a twice-daily dosing regime [41]. This dosing regime, particularly in oral form, would have considerable advantages over GSNO administration, as discussed above.

A phase 2 trial of the GSNOR inhibitor, N91115 (now named cavosonstat) has been performed in 138 adults with a homozygous F508del-CFTR mutation at doses of 200 and 400 mg bd compared to placebo. All participants were also receiving standard treatment with Orkambi (Vertex Pharmaceuticals, Boston, MA, USA), a combination of lumacaftor and ivacaftor. This trial has very recently reported and found no difference in the primary outcome, change in FEV1, across the groups (http://ir.nivalis.com/press-releases/detail/56). The future of GSNO reductase inhibitors in the treatment of CF is uncertain.

## Effect of GSNOR Inhibitors on Endothelial Function

Flow-mediated dilatation in pre-eclampsia has been shown to be consistently lower in pre-eclampsia compared to normotensive pregnancies [42–47], compatible with poorer endothelial function. In pre-eclamptic pregnancies complicated by IUGR, FMD is further reduced [44, 48]. FMD is a non-invasive marker of endothelial function and, as such, NO bioavailability. In pre-eclampsia, it has been suggested that alterations in FMD may be, in part, due to alterations in the downstream effect of cGMP rather than due to the availability of NO, per se [49].

The novel GSNO reductase inhibitor N6338, was tested in a hypertensive rat model (Dahl-S rats fed a high salt (4%NaCl) diet) and compared to a normotensive group. GSNO reductase activity was tested in both the heart and aortic tissue and was significantly reduced by the addition of N6338. Further in vitro studies showed that the addition of L-NMMA, an eNOS inhibitor, attenuated the effect of GSNO reductase inhibition in pre-constricted (using phenylephrine) aortic rings. This demonstrated the dependence of N6338 effects on the classic cGMP/NO model of smooth muscle relaxation. Flow-mediated dilatation was then tested 24 h after administration of N6338. This showed that the GSNOR inhibitor prevented an l-NMMA reduction in FMD. In the hypertensive rats, inhibition of GSNO reductase caused a fall in vascular resistance, a reduction in blood pressure and restored FMD to a level similar to normotensive rats [32]. Importantly, this study demonstrated reversal of hypertension-induced renal changes of hypertension: this would be a valuable therapeutic benefit in pre-eclampsia and accords with our finding of a reduction in proteinuria with GSNO infusion [38].

## Conclusion

Pre-eclampsia remains a significant cause of maternal and neonatal mortality and morbidity worldwide. Although of seizure prevention with MgSO4 and awareness of the need to detect and treat hypertension has made some headway into improving outcomes, there remains no treatment modality that targets the endothelial dysfunction that underlies that pathological process that manifests as pre-eclampsia.

The only drug currently under investigation that targets the NO pathway is sildenafil. To date the results of studies of sildenafil in pre-eclampsia have not demonstrated a benefit. Our group has demonstrated that intravenous infusion of GSNO has cardiovascular, platelet, and, possibly, renal effects on women with severe early pre-eclampsia that could be beneficial and could warrant further investigation. There are considerable difficulties in the preparation, administration, and refrigeration storage of GSNO.

The recent discovery and development of GSNO reductase inhibitors albeit in other therapeutic areas provides a promising option. Several of these which have passed cytotoxic and mutagen assessment and have proven themselves to be well tolerated in phase 1 studies. They lend themselves to use in the second and third trimesters of pregnancy. Animal studies showing improvement in endothelial function and hypertension with GSNO reductase inhibitors are consistent with our observations of the effect of GSNO in human. The particular advantage of GSNOR inhibitors is the ability to administer orally.

The inertia engendered because of concerns over drug development in pregnancy has in the longer term led to a therapeutic wilderness in which conditions potentially amenable to treatment are overlooked [2]. At least on a mechanistic level, GSNOR inhibitors offer a potentially promising option for the management of pre-eclampsia.
